# Effects of T-cadherin expression on B16F10 melanoma cells

**DOI:** 10.3892/ol.2013.1164

**Published:** 2013-01-30

**Authors:** XIN-SUO DUAN, JIE LU, ZHI-HUA GE, EN-HONG XING, HAI-TAO LU, LI-XIN SUN

**Affiliations:** 1 Departments of Dermatology, The Affiliated Hospital of Chengde Medical College, Chengde, Hebei 067000, P.R. China; 2 Stomatology, The Affiliated Hospital of Chengde Medical College, Chengde, Hebei 067000, P.R. China; 3 Central Laboratory, The Affiliated Hospital of Chengde Medical College, Chengde, Hebei 067000, P.R. China

**Keywords:** invasiveness, proliferation, apoptosis, T-cadherin, melanoma

## Abstract

Melanoma is one of the most deadly skin cancers. T-cadherin is an atypical member of the cadherin superfamily as it lacks the transmembrane and cytoplasmic domains and is anchored to cell membranes through glycosylphosphatidylinositol (GPI) anchors. T-cadherin downregulation is associated with a poorer prognosis in various carcinomas, such as lung, ovarian, cervical and prostate cancer, while in the majority of cancer cell lines, T-cadherin re-expression inhibits cell proliferation and invasiveness, increases susceptibility in apoptosis and reduces tumor growth in *in vivo* models. The functional relevance of T-cadherin gene expression in melanoma progression remains to be clarified. The present study was designed for this purpose. The T-cadherin gene was transfected into B16F10 melanoma cells to express T-cadherin in the cells which were originally deficient in T*-*cadherin expression. The proliferation, invasiveness, apoptosis and cell cycle of the transfected B16F10 melanoma cells were analyzed. The present study showed that the expression of T-cadherin in B16F10 melanoma cells markedly reduced cell proliferation and permeation through Matrigel-coated membranes, representing invasiveness. The percentage of early apoptotic cells and cells in the G_2_/M phase of the cell cycle was markedly increased compared with either parental B16F10 (without transfection) or empty pEGFP-N1 (without T-cadherin gene)-transfected B16F10 cells, suggesting G_2_/M arrest, with similarity between the parental and empty pEGFP-N1-transfected B16F10 cells. T-cadherin is important in melanoma progression and may be a possible target for therapy in melanoma and certain other types of cancer.

## Introduction

Melanoma is one of the most deadly skin cancers and the incidence of melanoma is continuing to increase world-wide (1). Cutaneous malignant melanoma (CMM) is the sixth most commonly diagnosed cancer in the USA in males and females (2). One person succumbs to from melanoma every hour in the USA. Advanced-stage cutaneous melanoma has a median survival time of <1 year. An important feature of melanoma is that the incidence rate is highest in lighter skinned patients and much lower in darker skinned individuals. Furthermore, melanoma strikes individuals before old age (median age, 52 years), almost a decade before the majority of solid tumors arise (e.g., breast, colon, lung or prostate). Metastasis is the main contributor to the high mortality. Once diagnosed with metastatic melanoma (AJCC stage IV), the majority of patients ultimately succumb to the disease within two years (3). The median survival following the onset of distant metastases is only six to nine months and the five-year survival rate is <5% (4).

One of the most significant factors associated with tumor invasiveness and metastasis is altered cell-cell adhesion, which determines cell polarity and is involved in cell differentiation, as well as the establishment and maintenance of tissue homeostasis. During oncogenesis, this organized adhesion is disturbed by genetic and epigenetic changes, resulting in changes in signaling, loss of contact inhibition and altered cell migration and stromal interactions. A major class of cell-cell adhesion molecules is the cadherin superfamily of transmembrane glycoproteins that typically mediate calcium-dependent homophilic intercellular adhesion (5). E-cadherin is a prototypic member of the cadherin superfamily and has been characterized as a potent suppressor of invasion and metastasis in studies dating back to the 1990s (6). Perturbations in cadherins have been associated with cancer, particularly invasion and metastasis (7). The decreased expression and abnormal cellular distribution of E-cadherin have been frequently observed to be associated with de-differentiation and invasiveness in a variety of human malignancies (8). T-cadherin (also known as CDH13 and H-cadherin) is an atypical cadherin superfamily member which is anchored in the membrane through a glycosylphosphatidylinositol (GPI) anchor instead of a transmembrane domain (9). T-cadherin has also been suggested to be involved in cancer progression (10,11). T-cadherin downregulation has been observed in various malignant tumors, including malignant melanoma (12), gallbladder carcinoma (13,14), ovarian carcinoma (15,16) and breast (17) and lung cancers (18,19). T-cadherin re-expression may suppress cell proliferation and invasiveness, increase sensitivity to apoptosis and decrease tumor growth (12,20–22).

The functional relevance of T-cadherin gene expression in melanoma progression remains to be further clarified. Our previous studies using B16F10 melanoma cells focused on the immune mechanisms and used *Ganoderma lucidum* polysaccharides to demonstrate the enhancement of MHC class I and costimulatory molecules in B16F10 cells (23), promotion of lymphocyte activation by B16F10 cells (24), antagonism against lymphocyte suppression caused by the culture super-natants of B16F10 melanoma cells (25), induction of marked cytotoxicity against B16F10 cells in cytotoxic T cells (CTLs) with granzyme B and porforin and inhibition of tumori-genesis in wild-type B16F10 melanoma cells *in vivo*(26). Cell proliferation, apoptosis, cell cycle and invasiveness are important features associated with malignancy (27). In the present study, the T-cadherin gene was expressed in B16F10 melanoma cells, which are deficient in T*-*cadherin expression, by T-cadherin gene transfection to examine its effects on proliferation, apoptosis, cell cycle and invasiveness in B16F10 melanoma cells.

## Materials and methods

### Cell culture system

The mouse B16F10 melanoma cell line, which is derived from C57BL/6 mice, was used in this study as it is originally deficient in T*-*cadherin expression. The B16F10 melanoma cell line was purchased from KeyGen Biotech. (Nanjing, China) and was cultured in RPMI-1640 medium supplemented with fetal bovine serum (10%), penicillin (100 IU/ml) and streptomycin (100 *μ*g/ml) at 37°C in humidified 5% CO_2_ atmosphere.

### Immunocytochemistry

The cells were cultured on slides and fixed with cold acetone for 5 min, then rinsed with distilled water. The endogenous peroxidase activity was quenched with 3% hydrogen peroxide. After blocking with 10% normal serum, rabbit polyclonal primary antibody against T-cadherin (Santa Cruz Biotechnology, Inc., Santa Cruz, CA, USA) was added at a 1:100 dilution and incubated overnight at 4°C. The next day, the horseradish peroxidase labelled secondary antibody was applied for 1 h and staining was finalized with a diaminobenzidine solution to detect the target antigen. Slides were extensively washed with phosphate-buffered saline (PBS) between the stages and counterstained with hematoxylin prior to mounting. The slides were examined under a light microscope. The replacement of primary antibody with PBS was used as a negative control.

### RNA extraction and RT-PCR

The total ribonucleic acids (RNAs) were extracted with TRIzol reagent (Invitrogen, Carlsbad, CA, USA) according to the manufacturer’s instructions. The RNA precipitates were dissolved in DEPC-treated double distilled water containing RNasin (R) ribonuclease inhibitor (Tiangen Biotech, Beijing, China). The concentration of RNA was determined spectrophotometrically (BioSpec-mini; Shimadzu, Kyoto, Japan). The messenger RNA (mRNA) of T-cadherin in the tissues for expression vector engineering was amplified by the reverse transcription polymerase chain reaction (RT-PCR) with an RT-PCR system (Tiangen Biotech) according to the manufacturer’s instructions. The levels of T-cadherin mRNA transcribed in the transfected cells were also detected by RT-PCR. To ensure that equal amounts of starting material were used in each RT-PCR reaction, RNA was reverse transcribed and amplified with β-actin-specific primers. The number of PCR amplification cycles was 34, to ensure that the amplification of all specific complementary deoxyribonucleic acid (cDNA) products was exponential. The specific primer sequences were as follows: T-cadherin sense primer, 5′-CCGGAATTCATGCAGCCGAG AACTCCGCT-3′ (for gene cloning) or 5′-TTCAGCAGAAA GTGTTCCATAT-3′ (for expression determination); T-cadherin antisense primer, 5′- CGCGGATCCTCACAGACA AGCTAAGCTGAAG-3′ (for gene cloning) or 5′-GTG CATGGACGAACAGAGT-3′ (for expression determination); β-actin sense primer, 5′-CCTCGCCTTTGCCGATCC-3′; and β-actin antisense primer, 5’-GACTGACTACCTCATGA AGATCC-3’. All of the products were electrophoresed on 2% agarose gel and stained with ethidium bromide. The expression intensities of the bands were quantified using ImageJ software (National Institutes of Health, Bethesda, MA, USA) and expressed as a ratio (T-cadherin vs. β-actin).

### Engineering of human T-cadherin expression vector and cell transfection

The pEGFP-N1/T-cad, a plasmid vector encoding human T-cadherin, was generated by inserting T-cadherin cDNA into a pEGFP-N1 vector (Clontech Laboratories, Inc., Mountain View, CA, USA), containing a CMV promoter and SV40 early promoter, as well as the neomycin/kanamycin resistance gene of Tn5 which allows stably transfected eukaryotic cells to be selected using G418. The vector backbone also contains an SV40 origin of replication in mammalian cells. The multiple cloning site (MCS) is next to the immediate early promoter of CMV (PCMV IE). The human T-cadherin gene was cloned from the mRNA extracted with TRIzol reagent (Invitrogen) from human uterine smooth muscle tissue by performing RT-PCR using the primers described previously in which the *Eco*RI or *Bam*HI restriction sites were included, digesting with *Eco*RI or *Bam*HI and ligating into the pEGFP-N1. The pEGFP-N1/T-cad was transfected into *Escherichia coli* TOP10 for amplification and DNA sequencing was used to ensure the fidelity. Plasmid DNA was prepared using the TIANprep Mini Plasmid Kit (Tiangen Biotech) as per the manufacturer’s instructions. Plasmids were transfected into the mouse melanoma cell line B16F10 cells at 80% confluence and 5×10^5^ cells per well in a six-well plate using the Lipofectamine™ 2000 (Invitrogen) according to the manufacturer’s instructions. The transfected cells which stably expressed T-cadherin were selected by incubating with G418 for >2 weeks. The stable expression of T-cadherin was confirmed by RT*-*PCR and immunohistochemistry.

### Cell proliferation assay

Cell proliferation was measured by the 3-(4,5-dimethylthiazol-2-yl)-2,5-diphenyltetrazolium bromide (MTT) assay following a 48 h incubation in 96-well microculture plates with a volume of 200 *μ*l/well. MTT solution (Sigma, St. Louis, MO, USA; 20 *μ*l, 5 mg/ml) was added to each well 4 h before termination. After 4 h of incubation, the cells were lysed and the purple formazan crystals were solubilized with DMSO. The plate was analyzed on a microtitre plate reader at 490 nm and the absorbance was translated into a cell proliferation ratio for comparison: Cell proliferation ratio = (test absorbance / non-transfection control absorbance) × 100.

### Detection of apoptosis via EGFP-annexin V and propidium iodide (PI) staining

Briefly, cells were cultured in six-well plates (Costar; Corning Inc., Corning, NY, USA) for 72 h at 37°C and 5% CO_2_. Cells (2×10^5^) were collected from each well and incubated with 5 *μ*l EGFP-annexin V and 5 *μ*l PI (KeyGen Biotech.) at room temperature for 15 min in the dark. Subsequently, 500 *μ*l annexin V binding buffer was added and flow cytometry was performed using a FACSCalibur flow cytometer (Becton Dickinson, Franklin Lakes, NJ, USA). Cells were considered to be apoptotic if they were annexin V^+^/PI^–^ (early apoptotic). Each analysis was performed using at least 10,000 events.

### Analysis of cell cycle using PI staining

Cells were cultured in 6-well plates (Costar; Corning Inc.) for 72 h at 37°C and 5% CO_2_. The cells were then harvested, washed with PBS and resuspended in 75% ethanol at 4°C overnight to make the membranes porous. After washing with PBS, the cells were incubated with 0.5 ml PI solution (5 mg PI, 2 mg RNase, 1 ml Triton X-100, 65 ml NS and 100 mg citrate sodium; volume to 100 ml, pH 7.4) for 30 min at 4°C in the dark to stain the nuclear DNA. The percentages of cells in the G_0_/G_1_, S and G_2_/M phases of the cell cycle were identified by FACSCalibur flow cytometry (Becton Dickinson) excluding cell doublets. The analyses were performed using CellQuest software (Becton Dickinson). Each analysis was performed using at least 10,000 events.

### Transwell invasion assay

*In vitro* invasion assays were performed using 8-*μ*m Transwell membranes (Corning Inc.) to measure tumor invasion. The Matrigel invasion chambers were prepared at 1:4 dilution and incubated for 1 h at 37°C and 5% CO_2_. Cells were washed with 1X PBS, resuspended in 0.1% fetal bovine serum (FBS)-RPMI-1640 and 2×10^5^ cells (200 *μ*l) were added to the Matrigel-coated upper chamber. RPMI-1640 culture medium containing 30% FBS was placed in the lower compartment of the chemotaxis chamber to function as a source of chemoattractants. The 24-well plastic culture plate was incubated at 37°C and 5% CO_2_ for 24 h. After incubation, the invasive cells which had passed through to the lower surface of the filter were fixed and stained with hematoxylin for microscope observation or quantified using an MTT assay in which the absorbance was translated into a cell ratio for comparison: Cell ratio = (test absorbance / non-transfection control absorbance) × 100.

### Statistical analysis

The results, with the exception of the immunocytochemistry, were expressed as the mean ± SD of triplicate experiments (six experiments in the MTT cell proliferation assay) and statistical comparisons between the experimental groups and the control were performed using one-way analysis of variance (ANOVA) followed by Dunnett’s t-test. P<0.05 was considered to indicate statistically significant differences.

## Results

### Confirmation of cDNA fidelity and expression in transfected melanoma cells

**Figure 1:**
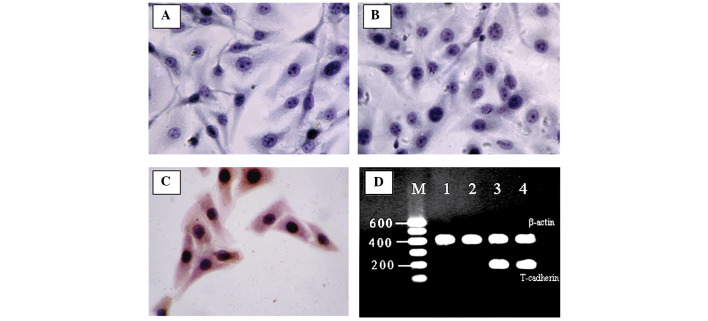
Demonstration of T-cadherin expression in T-cadherin-transfected B16F10 melanoma cells. B16F10 melanoma cells were transfected with the pEGFP-N1/T-cad plasmid vector encoding human T-cadherin to induce T-cadherin expression. After two weeks of culture with G418 for stable T-cadherin expression selection, immunocytochemistry and RT-PCR were used. Immunocytochemistry showing (A) negative T-cadherin expression in parental B16F10 cells (without transfection); (B) negative T-cadherin expression in empty pEGFP-N1 (without T-cadherin gene)-transfected B16F10 cells; and (C) positive T-cadherin expression in pEGFP-N1/T-cad-transfected B16F10 cells. (D) Electrophoresis on agarose gel and staining with ethidium bromide showing mRNA transcription in B16F10 cells detected by RT-PCR. (T-cadherin, 208 bp; β-actin, 435 bp); (1) control without transfection; (2) transfection of empty pEGFP-N1; (3) and (4) Transfection of pEGFP-N1/T-cad. RT-PCR, reverse transcription polymerase chain reaction.

The cDNA fidelity of the engineered human T-cadherin expression vector pEGFP-N1/T-cad was evaluated by DNA sequencing which showed that the cDNA was consistent with the genebank (NM_001257). The pEGFP-N1/T-cad was transfected into mouse B16F10 melanoma cells and the transfected cells which stably expressed human T-cadherin were selected by culturing with G418 for two weeks. The expression of T-cadherin was demonstrated by immunohisto-chemistry (Fig. 1A–C) and RT-PCR (Fig. 1D).

### Cell proliferation in T-cadherin-transfected melanoma cells

**Figure 2:**
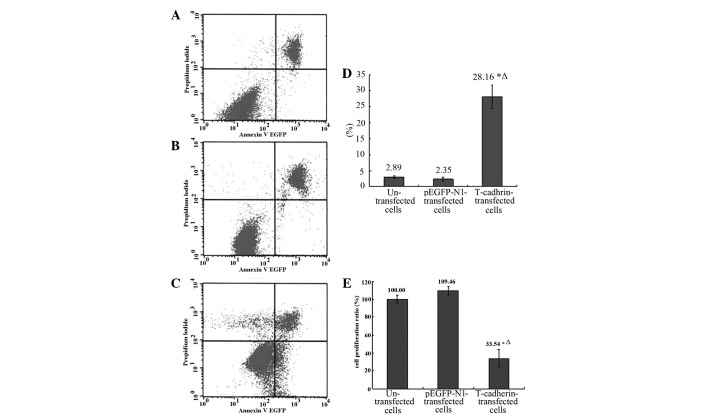
Proliferation and apoptosis in T-cadherin-transfected B16F10 melanoma cells. B16F10 melanoma cells were transfected with the pEGFP-N1/T-cad plasmid vector encoding human T-cadherin to induce T-cadherin expression. After two weeks of culture with G418 for stable T-cadherin expression selection, cells were cultured for 48 h, followed by an MTT assay to analyze proliferation and were cultured for 72 h followed by EGFP-annexin V/PI staining and flow cytometry analysis to evaluate apoptosis. Error bars indicate the standard deviation of the mean. ^*^Dunnett’s t-test P<0.05 compared with the parental B16F10 cells (without transfection) following one-way ANOVA. ^Δ^Dunnett’s t-test P<0.05 compared with the empty pEGFP-N1 (without T-cadherin gene)-transfected B16F10 cells following one-way ANOVA. Scatter graphs showing annexin V and PI staining in (A) parental B16F10 cells; (B) empty pEGFP-N1-transfected B16F10 cells; and (C) pEGFP-N1/T-cad-transfected B16F10 cells. (D) Mean percentages of B16F10 cells stained with annexin V without PI (early apoptotic cells). (E) Mean proliferation ratio in B16F10 cells (MTT assay). MTT, 3-(4,5-dimethylthiazol-2-yl)-2,5-diphenyltetrazolium bromide; PI, prop-idium idodide; ANOVA, analysis of variance.

It was shown by MTT assays that the proliferation in T-cadherin-transfected B16F10 melanoma cells was markedly lower than either the parental (without transfection) or empty pEGFP-N1 (without T-cadherin gene)-transfected B16F10 cells (P<0.05), while the proliferation in the parental and empty pEGFP-N1-transfected B16F10 cells was similar (P>0.05, Fig. 2E).

### Apoptosis in T-cadherin-transfected melanoma cells

Flowcytometry FACS analysis with annexin V and PI staining was used to determine cell apoptosis (28). It was shown by EGFP-annexin V/PI staining and flow cytometry that the percentage of early apoptotic cells in T-cadherin-transfected B16F10 melanoma cells was significantly larger compared with either the parental or empty pEGFP-N1-transfected B16F10 cells (P<0.05), while the percentage of early apoptotic cells in the parental and empty pEGFP-N1-transfected B16F10 cells was similar (P>0.05, Fig. 2A–D).

### Cell cycle in T-cadherin-transfected melanoma cells

**Figure 3:**
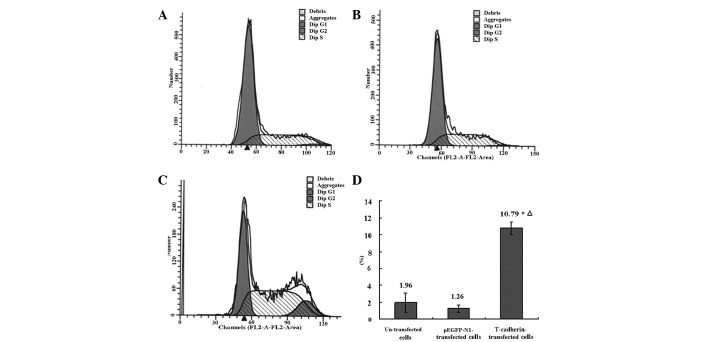
Cell cycle analysis in T-cadherin-transfected B16F10 melanoma cells. B16F10 melanoma cells were transfected with the pEGFP-N1/T-cad plasmid vector encoding human T-cadherin to induce T-cadherin expression. After two weeks of culture with G418 for selection of stable T-cadherin expression, PI staining and flow cytometry analysis were used. Error bars indicate the standard deviation of the mean. ^*^Dunnett’s t-test P<0.05 compared with the parental B16F10 cells (without transfection) following one-way ANOVA. ^Δ^Dunnett’s t-test P<0.05 compared with the empty pEGFP-N1 (without T-cadherin gene)-transfected B16F10 cells following one-way ANOVA. Histograms showing the cell cycle in (A) parental B16F10 cells; (B) empty pEGFP-N1-transfected B16F10 cells; and (C) pEGFP-N1/T-cad-transfected B16F10 cells. (D) Mean percentages of B16F10 cells in G_2_/M phase. PI, propidium idodide; ANOVA, analysis of variance.

The cell cycle may be analyzed by PI staining and flow cytometry analysis (29,30). It was shown by PI staining and flow cytometry analysis that the cell cycle in T-cadherin-transfected B16F10 melanoma cells was significantly different from either the parental or empty pEGFP-N1-transfected B16F10 cells. The cells in the G_2_/M phase were more abundant compared with either the parental or empty pEGFP-N1-transfected B16F10 cells (P<0.05), while the number of cells in the G_2_/M phase in the parental and empty pEGFP-N1-transfected B16F10 cells was similar (P>0.05, Fig. 3). This suggested that the T-cadherin-transfected B16F10 melanoma cells were arrested in the G_2_/M phase.

### Invasiveness of T-cadherin-transfected melanoma cells

**Figure 4:**
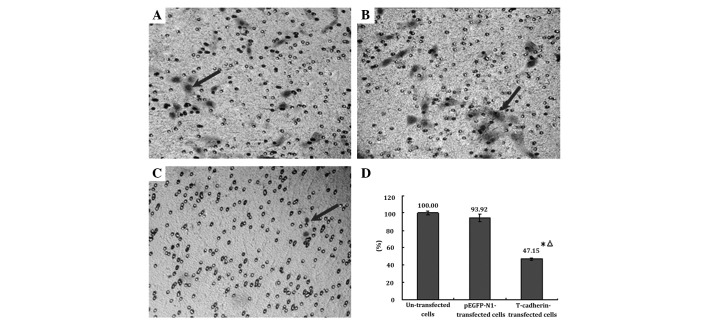
Invasiveness in T-cadherin-transfected B16F10 melanoma cells. B16F10 melanoma cells were transfected with pEGFP-N1/T-cad plasmid vector encoding human T-cadherin to induce T-cadherin expression. After two weeks culture with G418 for stable T-cadherin expression selection, the Transwell invasion assay was used with an MTT assay. Error bars indicate standard deviation of the mean. ^*^Dunnett’s t-test P<0.05 compared with parental B16F10 cells (without transfection) following one-way ANOVA. ^Δ^ Dunnett’s t-test P<0.05 compared with empty pEGFP-N1 (without T-cadherin gene)-transfected B16F10 cells following one-way ANOVA. Arrows identify invasive cells that had passed through to the lower surface of the filter in (A) parental B16F10 cells; (B) empty pEGFP-N1-transfected B16F10 cells; and (C) pEGFP-N1/T-cad-transfected B16F10 cells. (D) Mean cell ratio of invasive B16F10 cells that had passed through to the lower surface of the filter, as quantified by an MTT assay. MTT, 3- (4,5-dimethylthiazol-2-yl)-2,5-diphenyltetrazolium bromide; ANOVA, analysis of variance.

The Transwell assay is a useful method for determining cell migration (31) which represents invasiveness. It was shown by the Transwell invasion assay that the number of cells permeating the Matrigel-coated membranes in the T-cadherin-transfected B16F10 melanoma cells was significantly lower compared with either the parental or empty pEGFP-N1-transfected B16F10 cells (P<0.05). The number of cells permeating the Matrigel-coated membranes in the parental and empty pEGFP-N1-transfected B16F10 cells was similar (P>0.05, Fig. 4).

## Discussion

Invasiveness and metastasis are the most important features of malignant tumors, including melanoma. Cell adhesion is closely associated with tumor invasiveness and metastasis and the cadherin superfamily is a superfamily of transmembrane glycoproteins that mediate calcium-dependent homophilic intercellular adhesion. E-cadherin is a prototypic member of the cadherin superfamily and is expressed by the majority normal epithelial tissues and a number of epithelium-derived cancer cells have lost E-cadherin expression (32–34).

Unlike other members of the cadherin superfamily which contain transmembrane domains linking the extracellular portion of the molecules with the intracellular signaling pathways, T-cadherin is unique as it lacks the transmembrane and cytoplasmic domains and is anchored to cell membranes through a GPI anchor (9). Cell adhesion mediated by classical cadherins depends on the binding between cadherin extracellular domains presented on the surfaces of opposing cells and is regulated through intracellular associations with β- and α-catenins which affect the dynamics of the actin-based cytoskeleton (35,36). T-cadherin shares the extracellular five cadherin repeats with other cadherins but lacks the trans-membrane and cytosolic domains and instead attaches to the membrane via a GPI anchor (9). T-cadherin has been localized within lipid rafts of the plasma membrane, is targeted to the apical surface in polarized epithelial cells and redistributed to the leading edge of migrating cells (37–39). T-cadherin mediates calcium-dependent cell adhesion and colocalizes with small trimeric G-proteins and Src family kinases in lipid rafts, where it may be involved in modulating signal transduction pathways (37,40,41). T-cadherin mediates calcium-dependent adhesion, although it is not concentrated at the cell-cell junctions of transfected cells in culture (9,42). Thus T-cadherin appears to be far less adhesive than classical cadherins and, consistent with its role in cell growth and migration, is likely to be involved in reversible and dynamic cell-cell adhesion/de-adhesion (43). T-cadherin has been shown to have diverse roles in physiology and pathophysiology, including as a negative guidance cue for motor axon projections, tumor suppressor factor in various types of cancer, atypical lipoprotein-binding protein and stimulator of angiogenesis (44–46). Hence, in various studies T-cadherin has been shown to be involved in the regulation of proliferation, apoptosis and angiogenesis in normal tissues, as well as tumor growth (47).

T-cadherin is widely expressed in the brain and cardiovascular system, but is absent or strongly depleted in a number of cell cancer lines (18,20,21,48). Downregulation of T-cadherin gene expression associated with promoter hyper-methylation has been frequently reported in breast, lung and colon carcinomas (49). T-cadherin mediates the activation of PI3K/Akt/GSK3β signaling which protects endothelial cells from oxidative stress-induced apoptosis (45). T-cadherin downregulation is associated with poorer prognoses in various carcinomas, such as lung, ovarian, cervical and prostate cancer, while in the majority of cancer cell lines, T-cadherin re-expression inhibits cell proliferation and invasiveness, increases susceptibility to apoptosis and reduces tumor growth in *in vivo* models. In contrast to the majority cancer cell lines, T-cadherin overexpression in endothelial cells promotes proliferation and migration and has a pro-survival effect (10).

In the present study, it was shown that inducing the expression of T-cadherin by transfecting the T-cadherin gene into B16F10 melanoma cells markedly reduced cell proliferation and permeation through the Matrigel-coated membranes, representing invasiveness. The percentage of early apoptotic cells and the cells in the G_2_/M phase of the cell cycle was markedly increased compared with either the parental or empty pEGFP-N1-transfected B16F10 cells, with similarity between the parental and empty pEGFP-N1-transfected B16F10 cells. These properties suggest that T-cadherin may have an important role in melanoma progression and be a possible target for therapy in melanoma and certain other types of cancer.
